# Supplementary material for: Dynamic memory to alleviate catastrophic forgetting in continual learning with medical imaging
Source: Nat Commun. 2021 Sep 28;12:5678. doi: 10.1038/s41467-021-25858-z (PMC8479083; doi:10.1038/s41467-021-25858-z)
Supplement: Supplementary file 2 — Supplementary Information [file 41467_2021_25858_MOESM2_ESM.pdf]

1 Supplementary

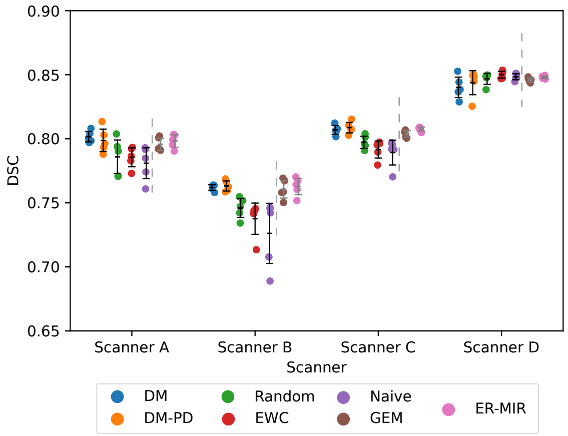

Supplementary Figure 1: Cardiac MR segmentation results after continual training measured as an average Dice score (DSC) over LV, RV, and MYO segmentation computed on the test set. Dice score for n=5 independent runs with different seeds are shown and error bars indicate mean (middle line) and standard deviation (whiskers). Dynamic memory (DM) is compared to DM with a pseudo-domain module (DM-PD), naive continual learning, random continual learning and EWC. In addition, GEM and ER-MIR are shown for reference, noting that they require information about domain membership.

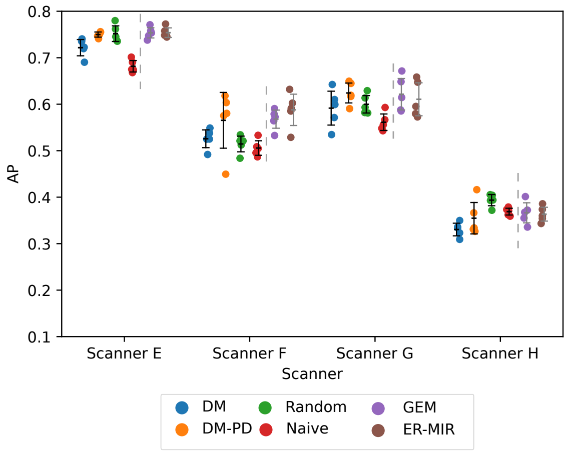

Supplementary Figure 2: CT Lung nodule detection results after continual training measured in average precision (AP) computed on the test set. AP for n=5 independent runs with different seeds are shown and error bars indicate mean (middle line) and standard deviation (whiskers). Dynamic memory (DM) is compared to DM with a pseudo-domain module (DM-PD), naive continual learning and random continual learning. In addition, GEM and ER-MIR are shown for reference, noting that they require information about domain membership.

Meth.	M	Scanner A	Scanner B	Scanner C	Scanner D	BWT	FWT
DM	128	0.854 ± 0.009	0.783 ± 0.010	0.835 ± 0.011	0.884 ± 0.011	−0.002 ± 0.003	0.022 ± 0.006
DM-PD	128	0.849 ± 0.013	0.789 ± 0.009	0.839 ± 0.004	0.892 ± 0.010	0.004 ± 0.004	0.020 ± 0.006
Random	128	0.846 ± 0.015	0.780 ± 0.008	0.832 ± 0.003	0.894 ± 0.005	−0.002 ± 0.005	0.022 ± 0.005
EWC		0.851 ± 0.003	0.778 ± 0.005	0.827 ± 0.006	0.902 ± 0.006	−0.005 ± 0.003	0.021 ± 0.003
Naive		0.849 ± 0.005	0.776 ± 0.006	0.828 ± 0.003	0.899 ± 0.006	−0.006 ± 0.003	0.020 ± 0.003
GEM	128	0.848 ± 0.003	0.791 ± 0.012	0.837 ± 0.003	0.892 ± 0.002	−0.004 ± 0.005	0.023 ± 0.003
ER-MIR	128	0.849 ± 0.003	0.788 ± 0.009	0.837 ± 0.007	0.893 ± 0.007	−0.007 ± 0.004	0.025 ± 0.004
DSM		0.845 ± 0.015	0.776 ± 0.014	0.843 ± 0.024	0.888 ± 0.007	-	-
JModel		0.860 ± 0.007	0.820 ± 0.019	0.850 ± 0.009	0.898 ± 0.010	-	-

Supplementary Table 1: Results for left ventricle segmentation (cardiac segmentation). Dynamic memory (DM) is compared to DM with a pseudo-domain module (DM-PD), random replacement strategy (Random), elastic weight consolidation (EWC) and naive continual learning (Naive). Methods requiring domain membership knowledge are gradient episodic memory (GEM), and experience replay with maximally inferred retrieval (ER-MIR). Domain specific models (DSM) and a joint model (JModel) serve as reference.

Meth.	M	Scanner A	Scanner B	Scanner C	Scanner D	BWT	FWT
DM	128	0.750 ± 0.011	0.704 ± 0.003	0.773 ± 0.007	0.811 ± 0.008	−0.008 ± 0.004	0.044 ± 0.007
DM-PD	128	0.751 ± 0.014	0.706 ± 0.008	0.774 ± 0.007	0.816 ± 0.012	−0.004 ± 0.006	0.045 ± 0.006
Random	128	0.734 ± 0.015	0.698 ± 0.005	0.759 ± 0.004	0.823 ± 0.008	−0.021 ± 0.005	0.051 ± 0.002
EWC		0.743 ± 0.010	0.693 ± 0.005	0.751 ± 0.008	0.825 ± 0.007	−0.018 ± 0.005	0.050 ± 0.004
Naive		0.741 ± 0.008	0.692 ± 0.006	0.753 ± 0.009	0.824 ± 0.0	−0.017 ± 0.006	0.049 ± 0.004
GEM	128	0.750 ± 0.006	0.708 ± 0.003	0.770 ± 0.008	0.826 ± 0.004	−0.010 ± 0.003	0.052 ± 0.005
ER-MIR	128	0.849 ± 0.009	0.788 ± 0.005	0.837 ± 0.002	0.893 ± 0.006	−0.007 ± 0.002	0.025 ± 0.004
DSM		0.759 ± 0.022	0.705 ± 0.012	0.759 ± 0.010	0.810 ± 0.008	-	-
JModel		0.778 ± 0.014	0.750 ± 0.023	0.784 ± 0.008	0.829 ± 0.011	-	-

Supplementary Table 2: Results for left ventricular myocardium segmentation (cardiac segmentation). Dynamic memory (DM) is compared to DM with a pseudo-domain module (DM-PD), random replacement strategy (Random), elastic weight consolidation (EWC) and naive continual learning (Naive). Methods requiring domain membership knowledge are gradient episodic memory (GEM), and experience replay with maximally inferred retrieval (ER-MIR). Domain specific models (DSM) and a joint model (JModel) serve as reference.

Meth.	M	Scanner A	Scanner B	Scanner C	Scanner D	BWT	FWT
DM	128	0.801 ± 0.011	0.799 ± 0.007	0.813 ± 0.005	0.825 ± 0.009	0.009 ± 0.007	0.030 ± 0.003
DM-PD	128	0.796 ± 0.009	0.794 ± 0.006	0.814 ± 0.009	0.824 ± 0.011	0.009 ± 0.005	0.028 ± 0.005
Random	128	0.778 ± 0.017	0.760 ± 0.024	0.802 ± 0.014	0.824 ± 0.011	−0.005 ± 0.012	0.028 ± 0.007
EWC		0.763 ± 0.021	0.742 ± 0.038	0.796 ± 0.015	0.823 ± 0.008	−0.020 ± 0.020	0.026 ± 0.005
Naive		0.752 ± 0.039	0.710 ± 0.079	0.787 ± 0.029	0.822 ± 0.006	−0.032 ± 0.038	0.026 ± 0.004
GEM	128	0.792 ± 0.016	0.785 ± 0.020	0.809 ± 0.011	0.821 ± 0.004	0.002 ± 0.014	0.026 ± 0.005
ER-MIR	128	0.796 ± 0.013	0.792 ± 0.014	0.815 ± 0.007	0.825 ± 0.008	0.004 ± 0.010	0.030 ± 0.006
DSM		0.802 ± 0.023	0.764 ± 0.018	0.814 ± 0.013	0.808 ± 0.016	-	-
JModel		0.827 ± 0.014	0.823 ± 0.014	0.834 ± 0.014	0.828 ± 0.011	-	-

Supplementary Table 3: Results for right ventricle segmentation (cardiac segmentation). Dynamic memory (DM) is compared to DM with a pseudo-domain module (DM-PD), random replacement strategy (Random), elastic weight consolidation (EWC) and naive continual learning (Naive). Methods requiring domain membership knowledge are gradient episodic memory (GEM), and experience replay with maximally inferred retrieval (ER-MIR). Domain specific models (DSM) and a joint model (JModel) serve as reference.

Meth.	M	Scanner A	Scanner B	Scanner C	Scanner D	BWT	FWT
DM	1024	0.815 ± 0.003	0.779 ± 0.009	0.813 ± 0.006	0.834 ± 0.005	0.007 ± 0.003	0.032 ± 0.004
DM-PD	1024	0.804 ± 0.004	0.778 ± 0.003	0.810 ± 0.005	0.827 ± 0.006	0.003 ± 0.002	0.029 ± 0.006
DM	512	0.815 ± 0.003	0.774 ± 0.005	0.808 ± 0.003	0.828 ± 0.007	0.005 ± 0.001	0.029 ± 0.005
DM-PD	512	0.807 ± 0.002	0.770 ± 0.007	0.813 ± 0.005	0.827 ± 0.007	0.003 ± 0.004	0.028 ± 0.003
DM	256	0.811 ± 0.008	0.771 ± 0.004	0.808 ± 0.003	0.835 ± 0.006	0.004 ± 0.003	0.031 ± 0.005
DM-PD	256	0.810 ± 0.004	0.766 ± 0.006	0.813 ± 0.007	0.837 ± 0.010	0.003 ± 0.003	0.031 ± 0.008
DM	128	0.802 ± 0.005	0.762 ± 0.002	0.807 ± 0.004	0.840 ± 0.009	0.000 ± 0.002	0.032 ± 0.004
DM-PD	128	0.799 ± 0.010	0.763 ± 0.004	0.809 ± 0.005	0.844 ± 0.010	0.003 ± 0.004	0.031 ± 0.005
DM	64	0.790 ± 0.008	0.759 ± 0.005	0.804 ± 0.004	0.841 ± 0.003	−0.005 ± 0.001	0.032 ± 0.005
DM-PD	64	0.795 ± 0.009	0.752 ± 0.004	0.802 ± 0.003	0.841 ± 0.004	−0.006 ± 0.006	0.031 ± 0.006
DSM		0.802 ± 0.017	0.748 ± 0.012	0.806 ± 0.014	0.835 ± 0.005	-	-
JModel		0.822 ± 0.010	0.798 ± 0.016	0.823 ± 0.006	0.852 ± 0.007	-	-

Supplementary Table 4: Cardiac MR segmentation results after continual training measured as an average Dice score (DSC) over LV, RV, and MYO segmentation computed on the test set. ± indicates the interval with five independent runs with different seeds. Dynamic memory (DM) is compared to DM with a pseudo-domain module (DM-PD), domain specific models (DSM), and a joint model (JModel).

Meth.	M	Scanner E	Scanner F	Scanner G	Scanner H	BWT	FWT
DM	883	0.752 ± 0.005	0.598 ± 0.013	0.628 ± 0.022	0.386 ± 0.013	0.033 ± 0.010	0.070 ± 0.029
DM-PD	883	0.749 ± 0.010	0.544 ± 0.020	0.630 ± 0.037	0.325 ± 0.017	0.040 ± 0.011	0.072 ± 0.022
DM	512	0.757 ± 0.012	0.604 ± 0.038	0.628 ± 0.039	0.378 ± 0.027	0.030 ± 0.019	0.086 ± 0.024
DM-PD	512	0.738 ± 0.008	0.561 ± 0.023	0.629 ± 0.048	0.340 ± 0.022	0.042 ± 0.018	0.051 ± 0.015
DM	256	0.744 ± 0.016	0.573 ± 0.029	0.596 ± 0.031	0.342 ± 0.021	0.016 ± 0.016	0.071 ± 0.024
DM-PD	256	0.736 ± 0.024	0.583 ± 0.037	0.603 ± 0.039	0.376 ± 0.034	0.032 ± 0.18	0.070 ± 0.015
DM	128	0.722 ± 0.020	0.526 ± 0.021	0.592 ± 0.041	0.330 ± 0.015	0.030 ± 0.018	0.063 ± 0.016
DM-PD	128	0.750 ± 0.006	0.565 ± 0.067	0.624 ± 0.024	0.355 ± 0.038	0.028 ± 0.019	0.066 ± 0.030
DM	64	0.717 ± 0.019	0.503 ± 0.035	0.579 ± 0.029	0.323 ± 0.025	0.009 ± 0.020	0.066 ± 0.029
DM-PD	64	0.730 ± 0.014	0.543 ± 0.025	0.627 ± 0.070	0.352 ± 0.029	0.018 ± 0.018	0.067 ± 0.028
DSM		0.653 ± 0.047	0.441 ± 0.074	0.643 ± 0.067	0.454 ± 0.096	-	-
JModel		0.716 ± 0.063	0.522 ± 0.114	0.711 ± 0.058	0.419 ± 0.087	-	-

Supplementary Table 5: CT Lung nodule detection results after continual training measured in average precision (AP) computed on the test set. ± indicates the interval with five independent runs with different seeds. Dynamic memory (DM) is compared to DM with a pseudo-domain module (DM-PD), naive continual learning, domain specific models (DSM), and a joint model (JModel).
