# Supplementary material for: Dynamic memory to alleviate catastrophic forgetting in continual learning with medical imaging
Source: Nat Commun. 2021 Sep 28;12:5678. doi: 10.1038/s41467-021-25858-z (PMC8479083; doi:10.1038/s41467-021-25858-z)
Supplement: Supplementary file 3 — Reporting Summary [file 41467_2021_25858_MOESM3_ESM.pdf]

nature research

Corresponding author(s): Georg Langs

Last updated by author(s): Aug 12, 2021

Reporting Summary

Nature Research wishes to improve the reproducibility of the work that we publish. This form provides structure for consistency and transparency in reporting. For further information on Nature Research policies, see our Editorial Policies and the Editorial Policy Checklist.

Statistics

For all statistical analyses, confirm that the following items are present in the figure legend, table legend, main text, or Methods section.

n/a	Confirmed	
☐	☒	The exact sample size (n) for each experimental group/condition, given as a discrete number and unit of measurement
☐	☒	A statement on whether measurements were taken from distinct samples or whether the same sample was measured repeatedly
☒	☐	The statistical test(s) used AND whether they are one- or two-sided<br>*Only common tests should be described solely by name; describe more complex techniques in the Methods section.*
☒	☐	A description of all covariates tested
☒	☐	A description of any assumptions or corrections, such as tests of normality and adjustment for multiple comparisons
☐	☒	A full description of the statistical parameters including central tendency (e.g. means) or other basic estimates (e.g. regression coefficient) AND variation (e.g. standard deviation) or associated estimates of uncertainty (e.g. confidence intervals)
☒	☐	For null hypothesis testing, the test statistic (e.g. F, t, r) with confidence intervals, effect sizes, degrees of freedom and P value noted<br>*Give P values as exact values whenever suitable.*
☒	☐	For Bayesian analysis, information on the choice of priors and Markov chain Monte Carlo settings
☒	☐	For hierarchical and complex designs, identification of the appropriate level for tests and full reporting of outcomes
☒	☐	Estimates of effect sizes (e.g. Cohen's d, Pearson's r), indicating how they were calculated

Our web collection on statistics for biologists contains articles on many of the points above.

Software and code

Policy information about availability of computer code

Data collection	No software was used for data collection.
Data analysis	Custom Python (Version 3.6) code was developed for this research and is available at https://github.com/cirmuw/dynamicmemory. PyTorch 1.6.0 is used as a deep learning library.

For manuscripts utilizing custom algorithms or software that are central to the research but not yet described in published literature, software must be made available to editors and reviewers. We strongly encourage code deposition in a community repository (e.g. GitHub). See the Nature Research guidelines for submitting code & software for further information.

Data

Policy information about availability of data

All manuscripts must include a data availability statement. This statement should provide the following information, where applicable:

- Accession codes, unique identifiers, or web links for publicly available datasets
- A list of figures that have associated raw data
- A description of any restrictions on data availability

Data is publicly available:
Multi-Centre, Multi-Vendor & Multi-Disease Cardiac Image Segmentation Challenge (M&Ms), https://www.ub.edu/mnms/
LIDC-IDRI Lung Cancer, https://wiki.cancerimagingarchive.net/display/Public/LIDC-IDRI
LNDb Lung Cancer Challenge, https://lndb.grand-challenge.org/Data/

Field-specific reporting

Please select the one below that is the best fit for your research. If you are not sure, read the appropriate sections before making your selection.

- [x] Life sciences
- [ ] Behavioural & social sciences
- [ ] Ecological, evolutionary & environmental sciences

For a reference copy of the document with all sections, see nature.com/documents/nr-reporting-summary-flat.pdf

Life sciences study design

All studies must disclose on these points even when the disclosure is negative.

Sample size	Two independent experiments were performed:<br>1. Cardiac segmentation in MR imaging data: 320 patients.<br>2. Lung nodule detection in CT imaging data: 644 patients<br>The data sets used were chosen to reflect various domain shifts that need to be countered during continual learning.
Data exclusions	For cardiac segmentation no data were excluded.<br>For lung nodule detection from LIDC we exclude patients scanned on scanners different from the three most common settings (GE MEDICAL SYSTEM with low frequency kernel, GE MEDICAL SYSTEM with high frequency kernel and Siemens with B30f kernel. From LNDb data we excluded data with a lesion size <3mm to match the definition within LIDC.
Replication	We developed a continual learning method that is reproducible across data sets and imaging modalities.<br>To demonstrate replication of the method we performed two independent experiments:<br>1. Cardiac segmentation in MR imaging: Data from four scanner vendors (Siemens, GE, Philips, Canon) were used to perform cardiac segmentation.<br>2. Lung nodule detection in CT imaging: Data acquired with four different scanner settings are used to perform lung nodule detection.
Randomization	Images are split into training and testing data sets at random. The sequence of the images in the data stream is randomly sampled.
Blinding	No blinding is required due to the retrospective nature of the study.

Reporting for specific materials, systems and methods

We require information from authors about some types of materials, experimental systems and methods used in many studies. Here, indicate whether each material, system or method listed is relevant to your study. If you are not sure if a list item applies to your research, read the appropriate section before selecting a response.

Materials & experimental systems

n/a	Involved in the study	
☒	☐	Antibodies
☒	☐	Eukaryotic cell lines
☒	☐	Palaeontology and archaeology
☒	☐	Animals and other organisms
☒	☐	Human research participants
☒	☐	Clinical data
☒	☐	Dual use research of concern

Methods

n/a	Involved in the study	
☒	☐	ChIP-seq
☒	☐	Flow cytometry
☒	☐	MRI-based neuroimaging
